# A comprehensive assessment of mangrove species and carbon stock on Pohnpei, Micronesia

**DOI:** 10.1371/journal.pone.0271589

**Published:** 2022-07-21

**Authors:** Victoria L. Woltz, Elitsa I. Peneva-Reed, Zhiliang Zhu, Eric L. Bullock, Richard A. MacKenzie, Maybeleen Apwong, Ken W. Krauss, Dean B. Gesch

**Affiliations:** 1 U.S. Geological Survey National Center, Reston, Virginia, United States of America; 2 The World Bank Group, Washington, DC, United States of America; 3 U.S. Geological Survey National Center, Reston, Virginia, United States of America; 4 Department of Earth & Environment, Boston University, Boston, Massachusetts, United States of America; 5 USDA Forest Service Pacific Southwest Research Station, Albany, California, United States of America; 6 Department of Biology, University of Hawaii, Honolulu, Hawaii, United States of America; 7 U.S. Geological Survey Wetland and Aquatic Research Center, Gainesville, Florida, United States of America; 8 U.S. Geological Survey Earth Resources Observation and Science Center, Sioux Falls, South Dakota, United States of America; University of the Aegean School of Social Sciences, GREECE

## Abstract

Mangrove forests are the most important ecosystems on Pohnpei Island, Federated States of Micronesia, as the island communities of the central Pacific rely on the forests for many essential services including protection from sea-level rise that is occurring at a greater pace than the global average. As part of a multi-component assessment to evaluate vulnerabilities of mangrove forests on Pohnpei, mangrove forests were mapped at two points in time: 1983 and 2018. In 2018, the island had 6,426 ha of mangrove forest. Change analysis indicated a slight (0.76%) increase of mangrove area between 1983 and 2018, contrasting with global mangrove area declines. Forest structure and aboveground carbon (AGC) stocks were inventoried using a systematic sampling of field survey plots and extrapolated to the island using k-nearest neighbor and random forest species models. A gridded or wall to wall approach is suggested when possible for defining carbon stocks of a large area due to high variability seen in our data. The k-nearest neighbor model performed better than random forest models to map species dominance in these forests. Mean AGC was 167 ± 11 MgC ha^-1^, which is greater than the global average of mangroves (115 ± 7 MgC ha^-1^) but within their global range (37–255 MgC ha^-1^) Kauffman et al. (2020). In 2018, Pohnpei mangroves contained over 1.07 million MgC in AGC pools. By assigning the mean AGC stock per species per area to the map, carbon stock distributions were visualized spatially, allowing future conservation efforts to be directed to carbon dense stands.

## 1. Introduction

Mangrove forests are among the world’s most productive ecosystems and provide many critical ecosystem services to coastal communities such as storm protection; provision of fish and timber; recreation; soil accretion; and climate change mitigation through carbon sequestration [2–4]. The relationship between mangrove forests and communities that depend on them is threatened by accelerating land use change and sea-level rise (SLR). Nowhere are local populations more reliant on mangroves than in the western Pacific region, including the multi-island nation of the Federated States of Micronesia (FSM), where remote island subsistence economies are supported by goods harvested from mangroves [5]. Such mangrove ecosystems are currently being impacted by SLR where geocentric rates are approximately three times higher than the global mean (3 mm yr^-1^) [6].

Donato and others found that Micronesian mangroves covered only 12–13% of the island area but held up to a third of the total carbon stock, containing 2–8 times more carbon per land area than savanna and upland forest [7]. Biogeochemical processes in mangroves, which limit organic matter decomposition in the soil, facilitates the sequestration of large amounts of carbon that might otherwise find its way to the atmosphere as CO_2_ or CH_4_ [8,9]. While tropical typhoons can occur frequently in the Pacific regions, few hit Pohnpei because of its location, allowing mangrove forests to accumulate much biomass aboveground as well as contribute to overall carbon stocks [10,11]. Therefore, the island of Pohnpei is likely to be distinctive in this regard, and may serve as a microcosm to explore carbon market value for mangrove resources Pacific-wide [12]. It is imperative to have a full accounting of mangrove area distribution, change over time, species composition, and structure information such as carbon stock. Tree species distribution models (SDMs) can be used to map species location, offer important insight into complicated interactions between species and their environement, and provide spatially explicit aids for conservation research [13].

**Fig 1:**
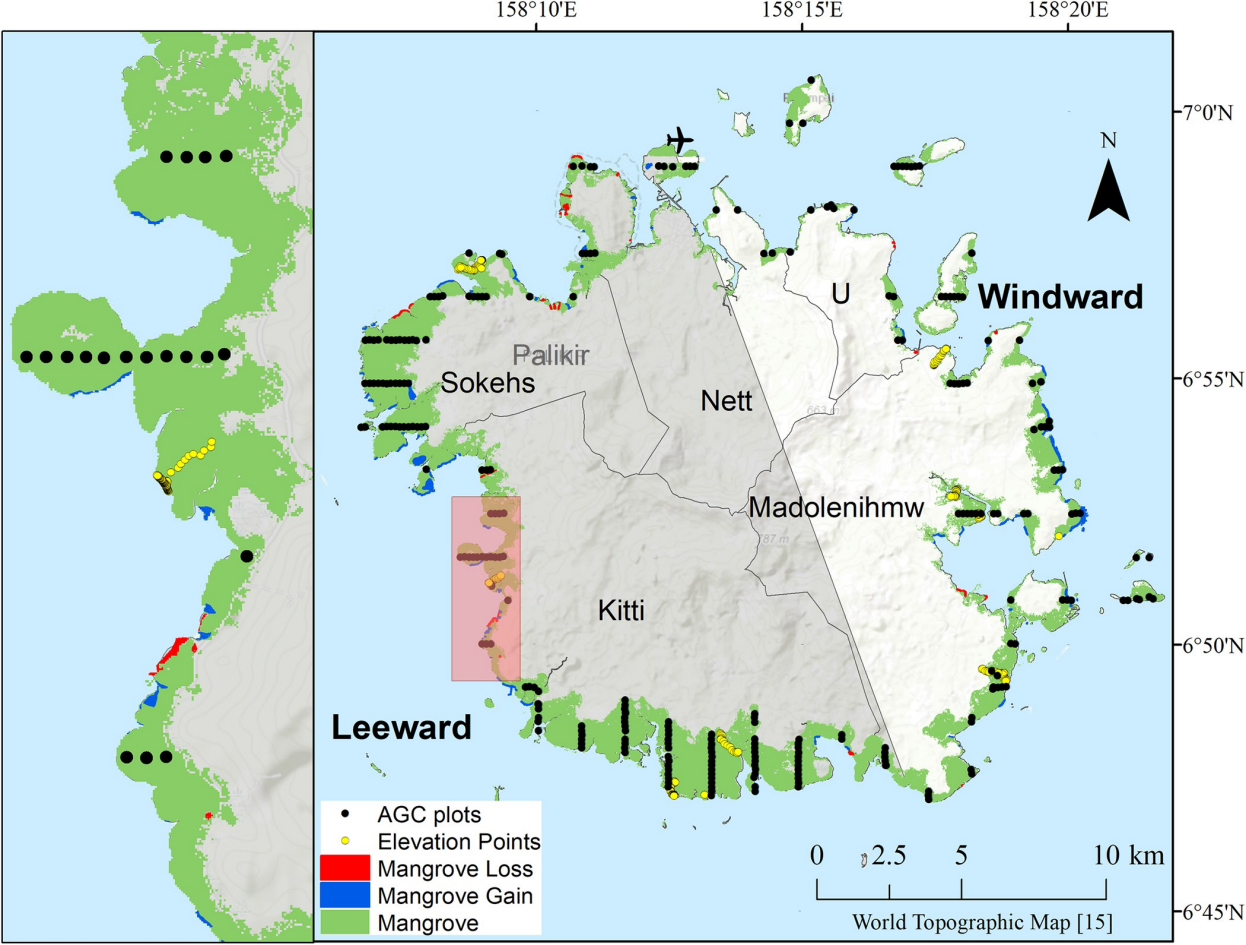
Mangrove area. Map of Pohnpei Island (right) and an insert for a portion of the island (left), showing areas of mangrove change (loss in red, gain in blue, and unchanged area in green) between 1983 and 2018 in 3 meter resolution. Locations of aboveground carbon (AGC) field survey plots; elevation points; municipality names; windward and leeward island sides (translucent white and grey, respectively); Pohnpei International Airport; and Palikir, the capitol of Federated States of Micronesia (FSM) are also shown on Esri’s topographic basemap [15]. In total, mangrove cover was 6,377 ha in 1983 and 6,426 ha in 2018, with a net change of plus 49 ha (loss 16, gain 65 ha).

Facing a disproportionately large impact from climate change, an initiative called Micronesia Challenge was created in 2006 to conserve near-shore marine and forest resources in Micronesia [14]. This spurred the launching of a major collaboration among U.S. Government, local government, non-governmental organizations, and community representatives to intensify sampling and conduct a comprehensive assessment of mangrove forests on Pohnpei, FSM (Fig 1), including remote sensing of mangrove extent and shoreline; SLR modeling; reviewing of existing permitting practices, regulations, and management regarding mangrove areas; and conducting a mangrove vulnerability evaluation. The main goal of the study described here was to characterize island-wide mangrove attributes, for the first time, with the specific objectives of: 1) producing a current mangrove map and determining change (loss and gain) of mangrove cover over recent decades, 2) mapping mangrove species using remote sensing modeling and field study, and 3) quantifying mangrove carbon stocks and distributions throughout the island.

## 2. Materials and methods

### 2.1.Study area

Pohnpei Island is located in the western Pacific (6° 53’ 31.6” N; 158° 12’ 52.9” E) and is surrounded by a fringing coral reef system 2–4 km from the coast [16]. Of the islands in FSM, Pohnpei is both the largest (334 km^2^) and the highest (790 m at the highest point) [16]. Pohnpei is a high, volcanic island characterized by a warm, breezy and humid tropical climate with a mean annual temperature of 31.9°C and an average annual lowland rainfall of 4,181 mm/yr which varies monthly [17–19]. Pohnpei is not commonly affected by typhoons since it is at the edge of the tradewind belt [20]. The mean wind speed is, on average, 5.53 m s^-1^ (10.75 knots), prevailing from 78° northeast, establishing the island’s east-northeast side as distinctively “windward” [21]. Mangrove forests border the island and comprise 15% of the land area [22]. Mangrove species (and/or putative hybrids) inventoried in our surveys included *Bruguiera gymnorhiza*, *Lumnitzera littorea*, *Rhizophora apiculata*, *R*. *x lamarckii*, *R*. *mucronata*, *R*. *stylosa*, *Sonneratia alba*, and *Xylocarpus granatum*. The mangrove species, *Pemphis acidula* and *Heritiera littoralis*, were not encountered during our surveys, though both have been recognized for their presence on the island [23].

### 2.2. Mangrove mapping and change analysis

Persistent cloud cover over Pacific tropical islands is a chronic issue for obtaining high quality satellite remote sensing data. A thorough search of the mid-resolution Landsat satellite imagery archive yielded no suitable scenes. However, a separate data search identified ten suitable high-resolution WorldView-3 satellite images from four days in 2018: July 3, September 28, October 17, and October 18. Image classification was conducted in Google Earth Engine (GEE) platform to derive a 2018 island-wide mangrove distribution map (Fig 1) and manual interpretation for extent change (see below). Image classifcation used the ten WorldView-3 images (calibrated to a 3 m resolution), a normalized difference vegetation index (NDVI) band calculated from each of the WorldView-3 images, and additional data products including digital elevation products (e.g. slope and aspect) derived from Shuttle Radar Topography Mission (SRTM). More details of the remote sensing methods used are described in S1 Appendix. The final map excluded inland non-mangrove land cover due to remaining heavy cloud cover, resulting in a 3 m resolution, island-wide mangrove map of 2018 draped on a base map of the island.

A set of 1983 aerial photographs of the island were further acquired, digitized and processed to form a complete island mosaic that matched dimension of the 2018 WorldView-3 image map. During the 1980s, the US Army Corps of Engineers contracted to have the coastal areas of many of the U.S.-affiliated Pacific Islands flown. As part of this assistance, large scale, color aerial photographs of Pohnpei’s coastline were taken in April of 1983. The photos were mosaicked in GEE to form an island-wide mosaic and resampled to 3 m resolution to match that of the 2018 WorldView-3 imagery. The result of the aerial photo processing was a 1983 mosaic image of three bands (red, green, blue) covering mangrove-occupied coastal areas of the island.

Controlling for quality problems, the two sets of the image data were analyzed in GEE for area changes (loss and gain), as described in S1 Appendix, resulting in a 1983–2018 mangrove loss and gain map for the island of Pohnpei (Fig 1). Difficult field conditions and time constraints on Pohnpei prevented collecting an additional, independent field dataset with which to measure accuracy of the maps. Instead, mapping performance was guided based on field and community knowledge of the mangrove forests of the island.

### 2.3. Field survey

On Pohnpei Island, FSM, a total of 273 plots were inventoried in 2016 and 2017 for forest structure and mangrove species; see Fig 1 for distribution of the plots. Plots were 10 m in radius with 3 m radius subplots. Protocol described in Peneva-Reed and others was followed for the field survey procedure, including sampling (systematic random sampling), determination of sample size, identification of tree species, mortality status, and measurements of live and dead trees as well as downed dead wood (see S1 Appendix for more information) [24]. Because *R*. *mucronata* and *R*. *stylosa* are difficult to distinguish without flowers and commonly occur in the same areas, these species were grouped together. There were also a few genus *Rhizophora* trees that could not be discerned and were kept separate in a *Rhizophora*-species unknown category. Aboveground plot data collected in the survey can be found in Peneva-Reed and others [25]. All protocols followed PLOS journal’s policy on inclusivity in global research (S3 Appendix).

### 2.4. Analyzing forest structure

Tree density, basal area, and importance value of tall and medium mangroves were quantified by species. Importance values were calculated as the sum of the relative density, relative basal area, and relative frequency and expressed as a percent. Density, basal area, and importance values of each species were also determined across seaward, interior and landward zones to analyze structural changes in the forest as distance from the sea increased. Kauffman and others define these three zones by their elevation [26]. For our paper, seaward plots were defined as being ≤ 30 m from the coast, landward plots were ≤ 30 m from the upland forest ecotone and interior plots were in between these. Our designation of “interior” would include a mix of the interior and riverine plot types defined previously for Micronesia [27]. Forest structure was also analyzed on the leeward (west-southwest) and windward (east-northeast) island sides, defined with knowledge of wind patterns from Bosserelle and others [21]. To gain insight into species habitat preference, seven two-sided, unpaired Student’s t-tests were run (one for each of the eight species minus one because *R*. *stylosa* and *R*. *mucronata* were combined) to compare their basal area by island side (p<0.05). The same test was used to compare species’ basal area by island zones, with only two zones being compared per test for a total of 21 tests (7 species times 3 combinations of zones). All t-tests were run in RStudio software [28].

### 2.5. Mapping dominant mangrove species

Non-parametric species distribution models (SDMs) offer important insight in understanding of habitat preferences and complicated species-predictor interactions and provide spatially explicit aids for conservation research and formulating policies [13,29]. Many studies choose to model species instead of community types because each tree has a specific spectral signature on the satellite images [30–32]. Dominant species, defined as species comprising the largest basal area per field plot, were analyzed separately with two geospatial model types: k-nearest neighbor (KNN) and random forest (RF) and a common set of predictor variables including principal components of satellite imagery composites, distance from water, elevation, and island side (leeward or windward) (see S1 Appendix for more details). Mangrove species models and resulting maps can be found in Peneva-Reed and others [25]. Due to heavy cloud cover, satellite image composites were used. The composites were comprised of WorldView-3 eight band images from July, September and October 2018; WorldView-2 three band image from December 2013; and Quickbird four band images from January 2007 and June 2005. The DEM was derived in ArcMap using the elevation points shown in Fig 1. Although many plots were sampled, when broken down into eight species, sample size for each species was much decreased.

After running the KNN model multiple times for testing purposes, it was found that sixteen nearest geospatial neighbors allowed for best model preformance. Model performance was measured using an approach of 10-fold cross validation with holdouts [33]. Model agreement was found by summing the plots where dominant species were correctly predicted and dividing by the total number of plots for each of the model runs and averaging them. After the cross-validation, the model algorithm created one map of all dominant species predictions.

One random forest model was created for each species for a total of eight RF models. RF models were performed in two ways; species data that had balanced dominant and nondominant plots were input into the randomForest function, while less balanced species data were input into the rf.classBalance function [34,35]. All RF models were run as classification with 1,000 trees without replacement and with ~36% out-of-bag samples. The sum of sensitivity and specificity was maximized to determine the threshold of each species being dominant [36]. Models were evaluated by determining their significance, running cross-validations and finding the performance metrics of back predictions (predicting values of the known plots using the created model). The 10-fold cross-validation was run 1,000 times, with 90% of the data used for training and 10% used for testing [36].

### 2.6. Aboveground biomass carbon from the field survey

Standing tree biomass was determined using allometric equations from similar world regions and DBH ranges when possible (Table A in S2 Appendix). Mortality status was recorded for all mangroves as follows: status one included recently dead trees with only leaves lost; status two included trees that had lost their secondary branches with only some primary branches remaining; and status three included trees that had lost secondary and primary branches and, typically, part of their main stem [37]. Biomass was estimated in dead status one trees by subtracting the biomass of the leaves from the whole tree, status two by subtracting 15% of the whole tree biomass, and status three as the biomass of only the main stem [37]. Allometric equations derived for *R*. *stylosa* were also used for *R*. *mucronata* because it is difficult to distinguish these two species in the field condition as they have similar form. *R*. *mucronata* could be misidentified as *R*. *stylosa* and vice versa. *Rhizophora x lamarckii*, a hybrid between *R*. *apiculata* and *R*. *stylosa*, is a relatively new species classification. Due to the lack of information on *R*. *x lamarckii* and a growth form more similar to *R*. *apiculata* than *R*. *stylosa*, allometric equations and specific gravity of *R*. *apiculata* was applied to *Rhizophora x lamarckii*. Species’ specific gravities used are listed in Hidayat and Simpson and Jachowski and others [38,39]. Standing tree carbon (STC) stock was estimated by multiplying biomass by the carbon conversion factor of 0.48 for live trees and 0.50 for dead trees [37,40].

Downed dead wood biomass was computed using the planar intercept technique with specific gravity from Micronesia [10,41]. Once carbon stock of each tree and downed wood piece on the forest floor was determined, they were summed for plots and divided by the plot area to get STC and downed wood carbon (DWC) stock per area. Average STC and DWC were added to get total aboveground carbon (AGC). Means ± standard errors are presented for carbon stock. To see which island side held more STC, a two-sided, unpaired Student’s t-test was used to compare STC on the windward and leeward side of the island (p<0.05). This test was repeated with the DWC. Similarly, STC and DWC were compared by zones (seaward, interior, and landward), with only two zones compared per test for a total of six tests (2 carbon sinks times 3 combinations of zones). All t-tests were run in RStudio software [28].

### 2.7. Upscaling carbon stock to Pohnpei

To determine total carbon stock in mangroves on Pohnpei, the forest strata approach was used by applying the mean AGC in all plots to mangrove area as determined by the 2018 high-resolution mangrove map [42]. Similarly, carbon gain and loss were calculated by multiplying the average AGC by the associated area. It is recognized that this method does not account for carbon gain in areas of undisturbed forests due to tree growth, nor does it precisely portray areas of gain and loss since lost mature mangroves would contain higher per-hectare carbon density than immature stands. Despite these drawbacks this method is common practice when historical data are not available. Carbon maps were created by assigning the amount of carbon in MgC ha^-1^ to the species maps. To compute carbon per hectare of a given dominant species, the mean carbon per tree ≥ 5 cm DBH of like species was averaged and multiplied by the average tree density of 890 ± 32 ha^-1^.

## 3. Results

### 3.1.Mangrove area and change

**Fig 2:**
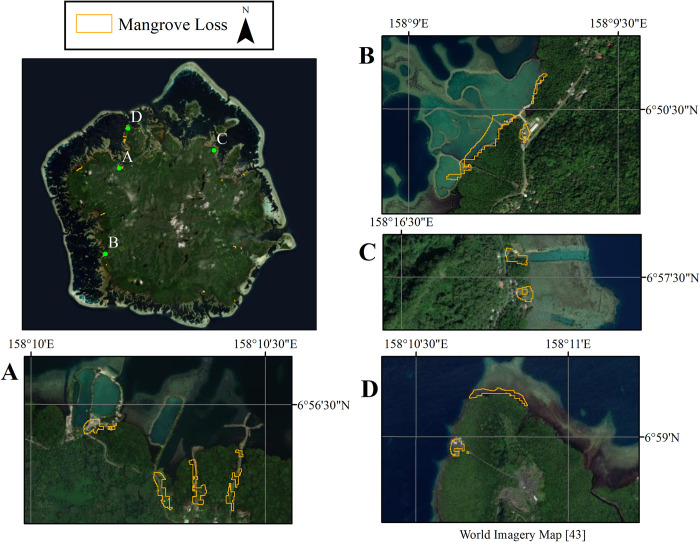
Mangrove loss. Areas of mangrove loss between 1983 and 2018 were primarily due to direct human activity such as building of roads, channels, commercial and residential areas, and industrial ponds (examples can be seen in images A-C). Other mangrove loss could be due to erosion (image D). Note images A-D correspond to green points in top left image. Satellite imagery seen in the figure is an Esri’s ArcGIS basemap [43].

In 1983, Pohnpei had 6,377 ha of mangrove, the total mangrove area gained 49 ha in 35 years, resulting in 2018 mangrove area of 6,426 ha (Fig 1). The 49-ha net gain was the result of 16 ha lost and 65 ha gross gain from various locations. While mangrove covered Pohnpei on all sides of its coasts (Fig 1), the forest was concentrated most in its leeward (west-southwest) side. It is on this side where mangrove forests lost and gained the most as well. In 2018, mangrove cover between the landward and seaward zones on Pohnpei had an average width of about 1,500 meters on the leeward side, and 400 meters on the windward (east-northeast) side. Area gained between 1983 and 2018 occurred primarily in the seaward zone of the forest, which is likely due to mangrove land building via sediment accrual in root systems and prevention of erosion from waves and subsequent growth of mangroves in response to newly accreting land forms [1,24]. Areas of mangrove loss were primarily in the seaward zone but also occurred frequently in the interior and landward zones. Loss was primarily due to human activity such as building of roads, channels, commercial and residential areas, industrial ponds, and harvesting of trees for firewood (Fig 2).

### 3.2. Dominant mangrove species

**Fig 3:**
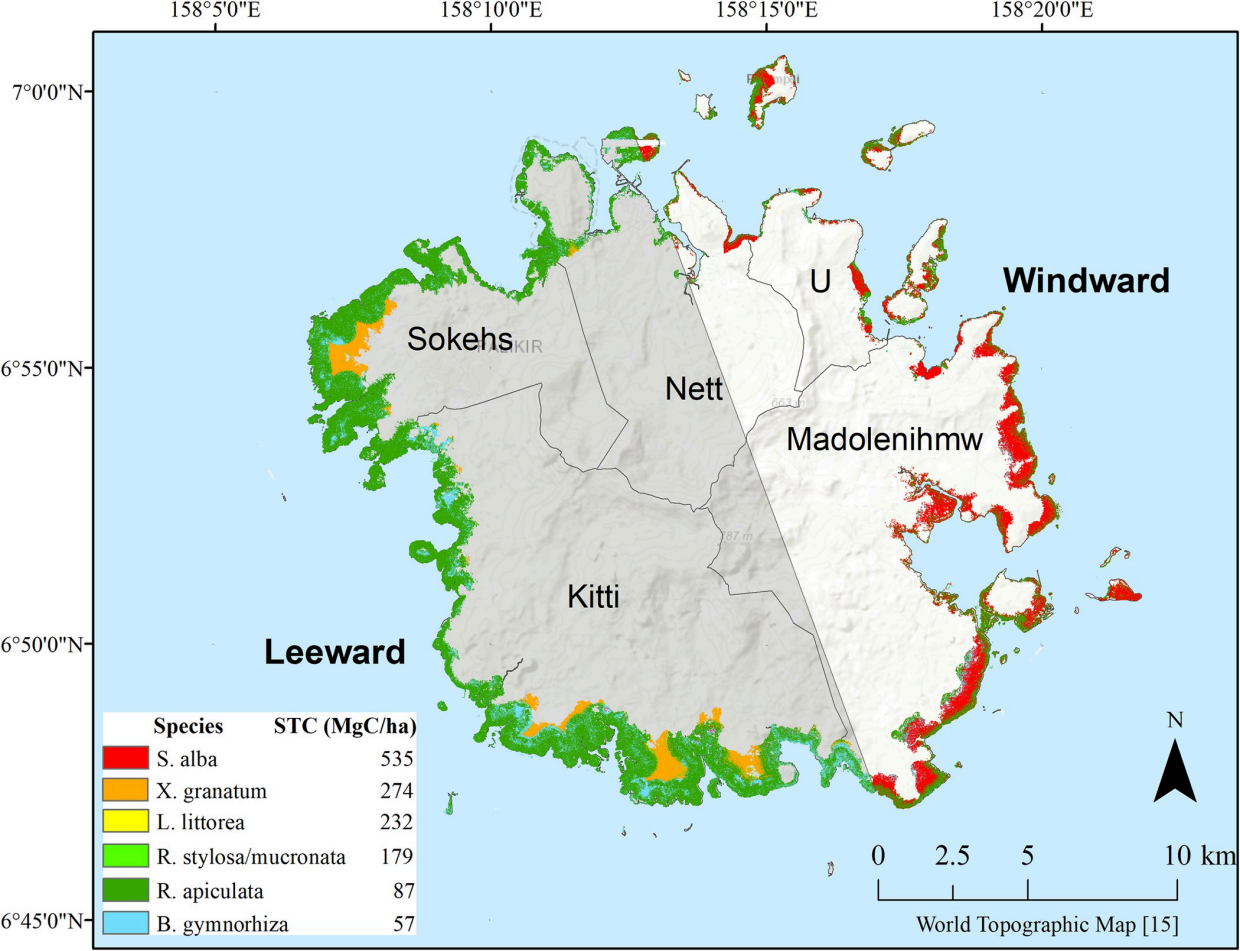
Dominant mangrove species. Dominant mangrove species and associated standing tree carbon (STC) stock (MgC ha^-1^) in 5 by 5 meter cells mapped using the k-nearest neighbor method, overlaid on Esri’s topographic basemap of the island of Pohnpei [15]. Municipality names and windward and leeward island sides (translucent white and grey, respectively) are also shown on the map.

Pohnpei dominant mangrove species distributions, based on the KNN model, are shown in Fig 3. *R*. *apiculata* was predicted to be dominant in 63% of the forest, with the remaining mangrove area mostly dominated by *S*. *alba*, *B*. *gymnorhiza*, and *X*. *granatum* (Table 1). Other species dominated less than 1% of the mangrove forest with some species not dominant anywhere. An overall agreement of 45% was obtained based on a 10-fold cross validation of the model performance.

**Table 1:** Predicted species dominance.

Species	% of Plots	% Area	
	Field Data	KNN	RF
*B*. *gymnorhiza*	29	11	35
*L*. *littorea*	3	0	5
*R*. *apiculata*	24	63	6
*R*. *mucronata*	7	0	1
*R*. *stylosa*	1	0	4
*R*. *x lamarckii*	3	0	3
*S*. *alba*	21	18	7
*X*. *granatum*	12	7	7
Total	100	100	66

The table shows the percent of plots a species was dominant in from the field data based off basal area and compares it to the percent of mangrove area that species were dominant in (also based off basal area) according to the models. Species were modeled simultaneously in the KNN model resulting in 100% of the forest area having one dominant species, but each species was modeled in a separate RF model resulting in some parts of the forest having more than one species or no species predicted to be dominant.

The KNN model outperformed the random forest model (details of RF performance in S1 Appendix and Table B and Fig A in S2 Appendix) based on field data (Table 1) and in field knowledge of the area. Though the KNN model underpredicts minority species, it predicted *B*. *gymnorhiza*, *R*. *apiculata* and *S*. *alba* as the three most common dominant mangrove species on Pohnpei as is true in the field data. Conversely, the RF model lowers the dominance of *R*. *apiculata and S*. *alba* to similar levels of more rare species in the field data such as *R*. *mucronata*, *R*. x *lamarckii*, and *L*. *littorea*.

### 3.3. Aboveground biomass and carbon

**Fig 4:**
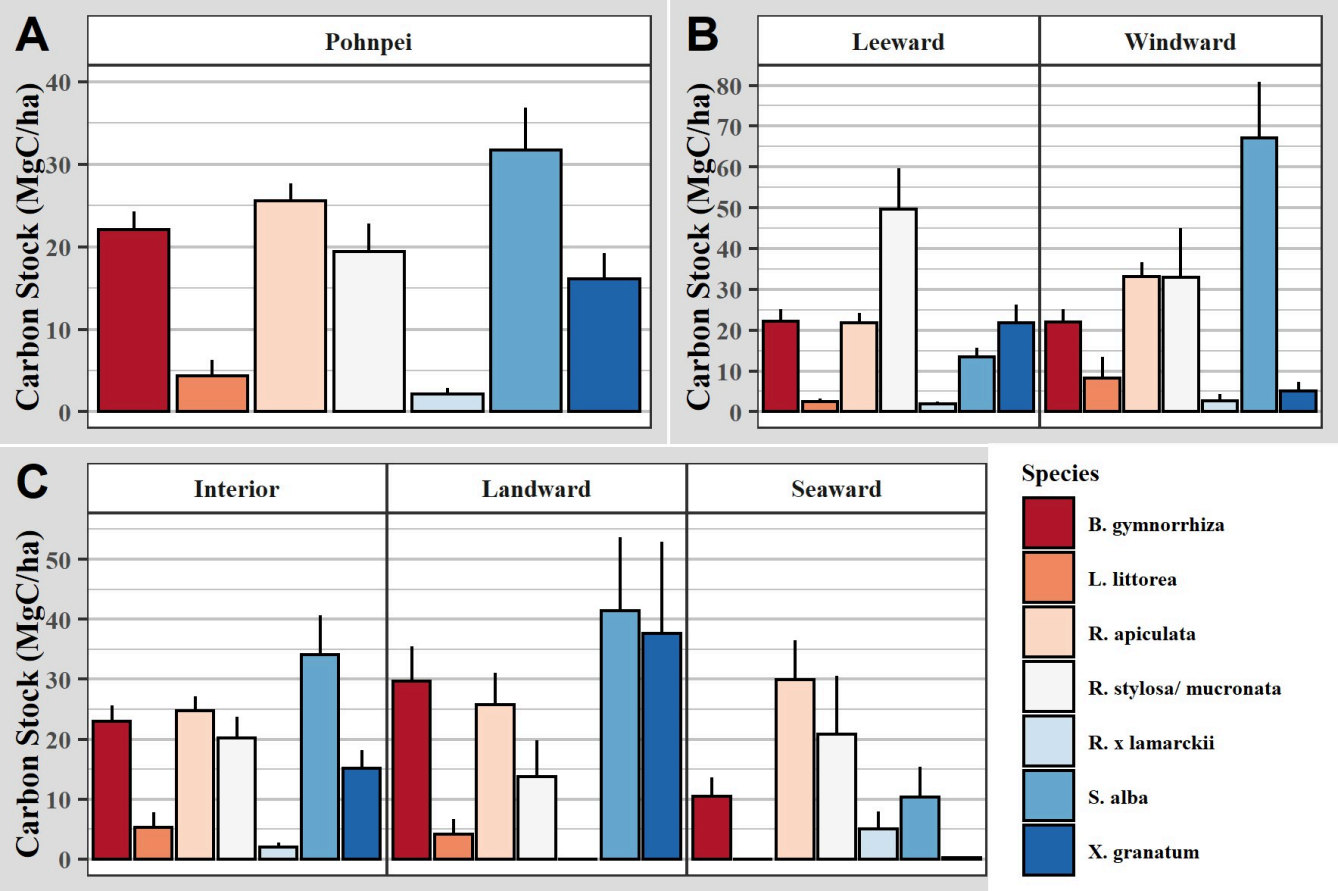
Carbon stock of mangrove species. Bar graphs showing mean and standard error of standing tree carbon stock (MgC ha^-1^) by dominant mangrove species on: A) Pohnpei Island-wide, B) island sides (windward vs leeward), and C) island zones (seaward, interior, vs landward). Island sides are shown in Fig 1 and zones are discussed in the text.

Out of the eight mangrove species studied on Pohnpei, *B*. *gymnorhiza* and *R*. *apiculata* had the highest importance values and were the densest species (Table 2). Total mangrove density in our plots varied from 32 to 3,247 trees ha^-1^ with a mean of 890 ± 32 trees ha^-1^. Total mean basal area was 39 ± 2 m^2^ ha^-1^. *S*. *alba* was not very dense but had the largest basal area of 11 ± 1 m^2^ ha^-1^ due to large mean DBH. Mean aboveground biomass and carbon were 344 ± 22 Mg ha^-1^ and 167 ± 11 MgC ha^-1^, respectively (Table 2). Total standing tree carbon (STC) ranged from 1 to 1,128 MgC ha^-1^ in the plots with a mean of 122 ± 7 MgC ha^-1^, accounting for 73% of AGC stock. *S*. *alba* contained the most carbon with 32 ± 5 MgC ha^-1^, followed by *R*. *apiculata*, and *B*. *gymnorhiza* with 26 ± 2 and 22 ± 2 MgC ha^-1^, respectively (Fig 4A). Carbon in downed wood (DWC) ranged from 0 to 436 MgC ha^-1^ in the plots, with a mean of 46 ± 4 MgC ha^-1^, accounting for 28% of the AGC. AGC ranged from 6–1,200 MgC ha^-1^ in the 236 plots with standing tree and downed dead wood information collected.

**Table 2:** Community composition.

	Count	Basal Area	Tree Density	IVI	Mean DBH	Max DBH	Biomass	Carbon stock
		m^2^ ha^-1^	trees ha^-1^	%	cm	cm	Mg ha^-1^	MgC ha^-1^
**Standing Trees**								
*B*. *gymnorhiza*	2,970	10.0 ± 0.7	346 ± 27	31	16 ± 0.2	161.1	46 ± 4	22 ± 2
*L*. *littorea*	144	2.1 ± 0.7	17 ± 5	3	31 ± 1.1	222.7	9 ± 4	4 ± 2
*R*. *apiculata*	2,233	6.1 ± 0.5	260 ± 19	24	15 ± 0.2	56.7	53 ± 4	26 ± 2
*R*. *stylosa/ mucronata*	829	3.2 ± 0.5	97 ± 17	11	18 ± 1.2	57.4	40 ± 7	19 ± 3
*R*. *x lamarckii*	538	0.6 ± 0.2	63 ± 17	4	10 ± 0.2	54.5	4 ± 1	2 ± 1
*S*. *alba*	452	10.7 ± 1.4	53 ± 7	16	41 ± 1.4	300.8	66 ± 11	32 ± 5
*X*. *granatum*	448	6.5 ± 1.1	52 ± 9	11	30 ± 1.2	230.8	33 ± 6	16 ± 3
All Species	7,636*	39.2 ± 1.9	890* ± 32	100	18 ± 0.2	300.8	253* ± 14	122* ± 7
**Downed Dead Wood**								
							91 ± 8	46 ± 4
**Aboveground Total**								
							344 ± 22	167* ± 11

The number, mean basal area (m^2^ ha^-1^), tree density (trees ha^-1^), importance value index (IVI) as a percent, mean and maximum DBH (cm), biomass (Mg ha^-1^) and carbon stock (MgC ha^-1^) of medium and tall mangrove species on Pohnpei, as well as biomass (Mg ha^-1^) and carbon stock (MgC ha^-1^) of down dead wood and total aboveground carbon. Values are presented as mean ± standard error except for in the case of IVI.

*Values do not add up because totals include unidentified *Rhizophora* species and/or because of rounding.

**Fig 5:**
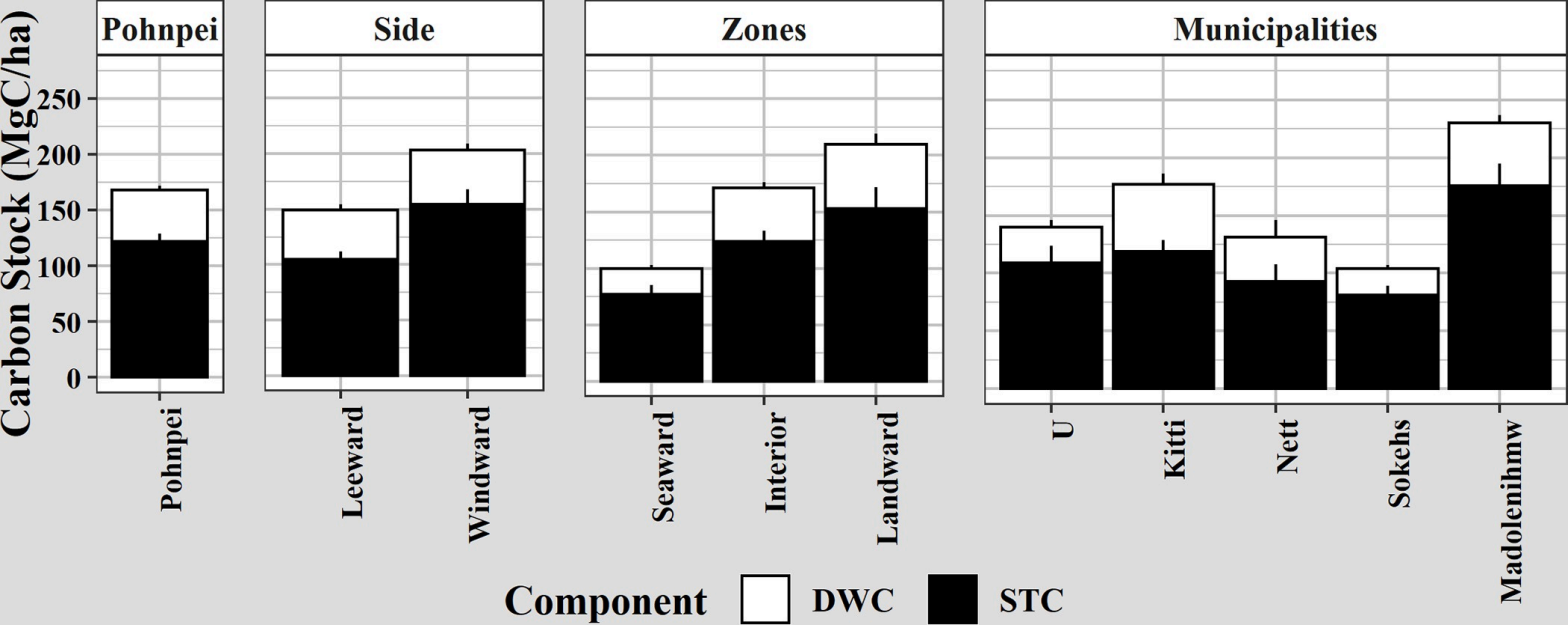
Carbon stock by location on Pohnpei Island. Bar graphs of mean and standard error of standing tree carbon (STC) and downed wood carbon (DWC) stock (MgC ha^-1^) by location of the field plots on the island of Pohnpei. Island sides are shown in Fig 1 and zones are discussed in the text.

Total basal area was higher on the windward (east-northeast) side of the island (see Fig 1), but mangroves were less dense. On the leeward (west-eouthwest) side of the island, *B*. *gymnorhiza* was twice as dense and was the most important species, with an importance value percentage of 34%, followed by *R*. *apiculata* with 22% (Table C in S2 Appendix). There was also more STC and DWC per forest area on the windward (east-northeast) side of the island (Fig 5) but only STC was significantly greater (p = 0.002). *S*. *alba* carbon was five-fold higher on the windward side and this species held the most carbon stock there. STC on the leeward (west-southwest) side was stored primarily in *R*. *stylosa/mucronata* followed by *B*. *gymnorhiza*, *R*. *apiculata*, and *X*. *granatum* with equal parts carbon (Fig 4B).

The landward zone was the least dense and had the most basal area due to the low disturbance (Table C in S2 Appendix). The highest tree densities were in the interior zones. The seaward zone had the lowest basal area and trees were denser than the landward zone. This suggests that trees are younger when they die in the seaward zone. This is likely due to high salinity and intense wave action from various sources. AGC increased as distance from the ocean increased with the landward zone containing more than twice as much AGC than the seaward zone (Fig 5). The seaward zone contained significantly less STC and DWC than both the interior and landward zones (p-values 7.71E-04 and smaller), but the interior and landward zones did not significantly differ. Most carbon stock in the seaward zone was in *R*. *apiculata*, while most carbon stock in the interior and landward zones was in *S*. *alba* (Fig 4C).

Madolenihmw, a municipality on the windward side of Pohnpei (Fig 1), contained the largest amounts of AGC per area, with 230 ± 26 MgC ha^-1^ (Fig 5). This is because *S*. *alba* contained the highest average carbon per tree and the most basal area on the windward side (Table C in S2 Appendix). The least amount of AGC per area was in Sokehs municipality with 104 ± 11 MgC ha^-1^.

### 3.4. Current total aboveground carbon stock and stock increase of mangroves since 1983

Aerial imagery showed that the mangrove area covered 6,426 ha in 2018. An estimated total of 1,073,142 MgC is stored aboveground in Pohnpei’s mangrove ecosystems. The carbon map made based off the KNN model (Fig 3) predicts species containing more carbon on the windward (east-northeast) side (primarily in *S*. *alba*) and species containing less carbon on the leeward (west-southwest) side (primarily in *R*. *apiculata*). Since 1983, 16 ha of mangrove forest have been lost, equating to 2,672 MgC, and 65 ha have been gained, equating to 10,855 MgC, for a net gain of 49 ha and 8,183 MgC in aboveground carbon of Pohnpei mangrove forest. This is likely an underestimate of carbon change as it does not include tree growth that occurred over this 35 year time period in intact stands.

## 4. Discussion

### 4.1.Mangrove change analysis

The results of mangrove mapping and change analysis (Fig 1) show that, over the 35 years of the analysis, mangrove loss occurred in various pockets mostly along the leeward (west-southwest) side. Anecdotal evidence indicates that the removal of mangrove cover along this side of the island has occurred as result of development in communities such as Sokehs and Kitti during the years of our change comparison (Fig 2). Land clearing for construction and farm creation leads to excess sedimentation downstream which can negatively impact mangroves [44]. It is speculated that loss of mangroves in Fig 2D could be due to upland construction, wave action, SLR or a combination of these factors. On Pohnpei, mangroves expanded more than retracted around all sides of the island but more so in the east (windward side) during the 35 years. This indicates the ability of mangrove species on Pohnpei (particularly *S*. *alba* in the windward side) to adapt to SLR by both expanding seaward as new sediments form adequate substrate elevations for mangrove colonization and also landward given opportunity. However, the ability of mangrove growth to keep pace with or exceed current SLR can be disrupted by construction of seaside infrastructure as seen in Fig 2 or accelerated SLR. Because of the high SLR rates, mangrove area gain and loss in and soil elevation of the seaward zone is of prime interest for future tracking [6]. Between 1975 and 2002, Pohnpei lost 70% of its original upland forest and most of this loss was to sakau (*Piper methysticum*) plantations [44]. Sakau is a mild narcotic grown on upland slopes for recreational use. It requires direct sun that comes from removal of surrounding canopy vegetation and has shallow roots that cause much erosion [45]. Significant erosion events that may occur only once after the first heavy rainfall after several dry months on other Micronesian islands, occur one to three times per month in some places on Pohnpei causing episodic high sediment loads in local streams [46]. Approximately 40% of this sediment is deposited on the mangrove ecosystems [46]. Though too much sedimentation can negatively impact mangroves, it is possible that continuous erosion of Pohnpei’s uplands could be creating more land for mangroves to colonize, explaining some of the increase in mangrove areas around the fringe of the island. Future studies of upland erosion and mangrove interactions on Pohnpei would be useful for characterizing these complex processes.

The change analysis compared two maps produced using different data sources (aerial photographs from 1983 and WorldView-3 satellite images from 2018), introducing uncertainties in the change information. Field plot data collected in the study were used as training data for mapping mangrove area. This left no separate, independent field data to assess map accuracies, although we were able to estimate species modeling performance with 10-fold cross-validation. To account for possible precision issues in shoreline change (which could lead to erroneous mangrove loss and gain), shoreline changes less than 10 m were discounted. However, the relatively small size of the island allowed for manual inspection of reference imagery for both the 2018 distribution map and change map. This mitigated possible map errors as much as possible. Furthermore, the only reference data that would be of higher quality than that used to make the map, and therefore suitable for accuracy assessment, would be random field points across the island which was not feasible for our surveys [47].

### 4.2. Species dominance models and carbon distribution

The KNN model visualized similar results to the field analyses of both carbon stocks and species location. Locating stands with dense carbon stock allows future conservation efforts to be directed accordingly. There was more carbon on the windward (east-northeast) side of the island with most of it being in the interior and landward zones. On the leeward (west-southwest) side of the island there was less carbon, with the least of it being in the seaward and interior zones.

In the nearby island of Kosrae, Ewel and others found no significant differences in porewater salinity and soil redox potential over basin and fringe zones and none of the mangrove species were restricted from any zone suggesting that hydrogeomorphic zonation could not be differentiated by species [27]. This study also found no true zonation of species, as species can and do inhabit a wide range of conditions. Dominance maps (based on in-situ basal area) do show preferences that were also seen in the field data (Table C in S2 Appendix) but with more spatially explicit detail. Allen and others found a tendency for *X*. *granatum* to associate with the landward edge, but it was not restricted to that area [48]. In agreement with field data, the KNN model predicted *X*. *granatum* dominance in the landward zone on the leeward side, while *S*. *alba* dominated landward and interior zones on the windward side. Field data showed that *R*. *apiculata* had more basal area in the seaward zone, which was shown in many areas in the KNN model. *R*. *stylosa*/*mucronata* often fringe waterways toward the ocean side of mangrove stands [49]. Similarly in Pohnpei, KNN map showed *R*. *stylosa*/*mucronata* along the seaward and interior zones on the leeward side of the island.

It is possible that some species could not be adequately modeled due to microclimatology, microtopography, and competition. Topography on relatively flat landscapes can be hard to model with fine detail but can play an intricate role in coastal species distributions. Competition, which was not modeled, could limit species from thriving in locations where conditions are otherwise favorable. In addition, some species have similar spectral band values when captured by satellite. Low spectral separability of species can limit the ability to model these species [50]. It is also likely that other predictors control mangrove distribution on Pohnpei that are not considered in our model, such as hydroperiods and nutrient availability [51]. Elevation can be a proxy for these predictors; however, the elevation points on Pohnpei used to interpolate a DEM surface were extremely sparse, increasing uncertainties in elevation (Fig 1).

### 4.3. Biomass and carbon stock

This study found aboveground biomass to be 344 ± 22 Mg ha^-1^ on Pohnpei Island. This is higher than both the estimated global mean (166 Mg ha^-1^) and Pacific Island mean (233 Mg ha^-1^) [52]. Our AGC mean was 167 ± 11 MgC ha^-1^, which is higher than the average of 115 ± 7 MgC ha^-1^ from multiple regions across the globe, but is still within the global range (37–255 MgC ha^-1^) (Table 3) [1]. AGC content on Pohnpei was similar to that in the Indo-Pacific region, containing an AGC mean of 159 MgC ha^-1^ [53]. Pohnpei’s mangrove AGC estimates were 36 MgC ha^-1^ higher than on Palau and 94 MgC ha^-1^ lower than on Yap [7]. The downed dead wood carbon percent of AGC on Pohnpei was higher than average and was most similar to that on Palau (Table 3). Pohnpei Island had significantly more STC per area on the windward side, however the opposite was found on Yap [10]. This is likely due to the intensification of tropical storms, when they do occur, as they pass from Pohnpei to Yap and to cause subsequent tree damage [20].

**Table 3:** Mangrove carbon stock from various locations world-wide.

	Standing Tree C		Downed Wood C		AGC	
Location	Mean ± SE	AGC %	Mean ± SE	AGC %	Mean ± SE	Reference
	MgC ha^-1^		MgC ha^-1^		MgC ha^-1^	
**Eastern Hemisphere**						
Pohnpei, FSM	122 ± 7	73%	46 ± 4	28%	167 ± 11	This Study
Asia	---	---	---	---	113 ± 12	[1]
Middle East	---	---	---	---	37 ± 8	[1]
South East	---	---	---	---	146 ± 14	[1]
Oceania	---	---	---	---	255 ± 22	[1]
West Africa	102 ± 10	91%	10 ± 1	9%	112 ± 12	[54]
West Central Africa	---	---	---	---	84+14	[54]
Micronesia, Indonesia and Bangladesh	---	---	---	---	159	[53]
Sulawesi, Indonesia	120 ± 12.7	---	---	---	---	[55]
Sumatra, Indonesia	173 ± 3*	---	---	---	---	[56]
Xishuangbanna, China	198 ± 30	96%	9 ± 2	4%	207 ± 32	[57]
Odisha, India	62 ± 6	---	---	---	---	[58]
Odisha, India	---	---	3.5 ± 0.6	---	---	[59]
Palau, FSM	101	77%	30	23%	131	[7]
Yap, FSM	249	95%	13	5%	261	[7]
**Western Hemisphere**						
Americas	---	---	---	---	83 ± 8	[1]
Central America	---	---	---	---	72 ± 8	[1]
South America	---	---	---	---	125 ± 15	[1]
Florida, USA	57 ± 1	85%	10 ± 1	15%	67 ± 2	[24]
Tabasco and Campeche, Mexico	121 ± 22	88%	17 ± 4	12%	138 ± 22	[1]
**Global**						
	---	---	---	---	115 ± 7	[1]

Aboveground carbon (AGC) values from different parts of the world in MgC ha^-1^. Standard error (SE) was reported when it was present in the reference material.

*Carbon stock calculated from biomass assuming that most biomass has a conversion factor of 0.48 [37].

By species, *S*. *alba* contained more aboveground carbon per area in Yap, FSM, Papua New Guinea, and the Philippines, but less in southwest India when compared to Pohnpei STC [10,60–62]. *R*. *apiculata* and *B*. *gymnorhiza* contained more carbon in Yap but less in the Philippines [10,61]. Many assessments that define mangrove carbon stock in entire countries or regions are based off few transects [1]. A gridded or wall to wall approach is suggested when possible for defining carbon stocks of a large area due to high variability seen in our data. When compared to other studies in Micronesia, our study found the widest range of aboveground biomass and carbon values per plot (13–2,494 MgC ha^-1^ and 6–1,200 MgC ha^-1^, respectively). In Yap, biomass and carbon ranged from 211–573 Mg ha^-1^ and 163–360 MgC ha^-1^, respectively and carbon in Palau ranged from 73–189 MgC ha^-1^ [7,10]. Our high variation in site carbon was likely due to the larger number of plots and greater overall area covered in our study. The amount of mangrove plots analyzed in our study was more than 4-fold higher than the other two aforementioned studies.

Mangrove plots on Pohnpei Island were established and researched in multiple studies prior to this one. In 2010 and 2011, Fujimoto and others found mangrove mean aboveground standing tree biomass (STB) of 658 Mg ha^-1^ in two plots in Madolenihmw, Pohnpei [63]. In this same municipality, our study found a mean STB of 366 ± 40 Mg ha^-1^ with a range from 2–2,349 Mg ha^-1^. These differences are likely due in part to the high level of variability in our data when intense spatial sampling is used. Allen and others reported an average downed dead wood volume of 43 m^3^ ha^-1^, which equates to 15 MgC ha^-1^ DWC in Madolenihmw, Pohnpei [20,40,41]. Our study found DWC to have mean of 46 ± 4 Mg ha^-1^ (range 0–436 MgC ha^-1^) in all Pohnpei sites and 54 ± 7 Mg ha^-1^ (range 0–357 Mg ha^-1^) in Madolenihmw. In 2003, there were two major storms, Super Typhoons Kujira and Lupit, the former being the more destructive with intense winds, tree felling, flooding and several deaths [64]. Because of the slow decomposition rates of mangroves, litter from these events has remained on the forest floor. The tropical storm that hit Pohnpei in May 2015, before moving on to become Typhoon Dolphin, caused $1 million (USD) in property damage alone and felled hundreds of trees [65] (p. 11). These three tropical storms hit Pohnpei after the studies by Allen and others and before our data collection, potentially leading to the higher DWC on Pohnpei in this study (Table 3) [20]. It is also possible that mangrove harvesting for fuel and construction has increased in recent years which would lead to more DWC if wood was unused or underutilized as on nearby Kosrae, FSM [20].

In addition to aboveground carbon and the change mapped for the island, belowground carbon contributes to the story as well. A recent unpublished study suggests that soils in Pohnpeian mangroves contain more than 6-fold higher carbon stock than the aboveground stocks, with 167 ± 11 MgC ha^-1^ in AGC and 1,150 ± 59 MgC ha^-1^ (range 681–1762) in soils to 2 meters in depth [M. Apwong, University of Hawaii, personal communication, 2019]. Other studies of mangrove belowground carbon storage on Pohnpei estimated 1,300 MgC ha^-1^ and 290 to 1,850 MgC ha^-1^ [66,67].

### 4.4. The future of Pohnpeian mangroves

Over the past 35 years (1983–2018), there was a slight (0.76%) increase in forested mangrove area on Pohnpei suggesting Pohnpei is still ahead of large-scale mangrove degradation providing an opportunity to develop regulations and community awareness proactively. Deforestation is an impending threat due to channel and road building, coral dredging, and residential encroachment such as clearing for landfills. Channels leading out to sea are being built to gain access to coral aggregate, used as building material, which is increasing in demand. There is also concern that forest will be replaced with aquaculture ponds due to decline in the local fishing industry and growing global demand for marine food. These forests are largely unimpacted currently giving Pohnpei the opportunity to raise community awareness and regulate these important ecosystems, before they are degraded and their ecosystem services lessened. The baseline maps of forest boundaries and carbon stock provided by this study, allowing for visual understanding of where resources are and what conditions they are in, can be useful for determining best management practices.

## 5. Conclusions

Ecosystem services such as carbon sequestration, habitat for fisheries, water filtering, and soil accretion make mangroves important from a management perspective. During the 1980s and 1990s mangrove forest area decreased by 50–80% in some regions and 35% worldwide primarily due, directly or indirectly, to increasing human population density [68]. South Asia and Asia-Pacific exhibits the highest global rates of mangrove loss [69]. However, forests on Pohnpei are still relatively intact and have seen a net area increase in recent years because many local residents value their benefits. However, SLR, channel building, coral dredging, development, and timber and fuelwood collection can cause deforestation and fragmentation inhibiting these ecosystems from providing services and creating carbon emissions. If conservation is to target carbon, efforts may benefit from a focus on Madolenihmw and Kitti municipalities as a start, because of the large amounts of carbon per area held in those mangrove forests. Land managers may consider policies that meet conservation goals and reflect local values to ensure the longevity of these forests and erosion protections for islanders [70]. Conservation funding could be achieved through potential carbon credit sales, either by payments to protect a carbon-heavy ecosystem, or by promoting conservation practices that facilitate habitat improvement or gain.

## Supporting information

S1 Appendix (DOCX)

S2 Appendix (DOCX)

S3 Appendix (DOCX)
