# Supplementary material for: A comprehensive assessment of mangrove species and carbon stock on Pohnpei, Micronesia
Source: PLoS One. 2022 Jul 21;17(7):e0271589. doi: 10.1371/journal.pone.0271589 (PMC9302747; doi:10.1371/journal.pone.0271589)
Supplement: S1 Appendix — (DOCX) [file pone.0271589.s001.docx]

**S1 Appendix**

1. **Methods supplement**
   1. **Mangrove mapping and change analysis**

The WorldView-3 multispectral imagery carried 8 spectral bands ranging from blue at 400 nm to a mid-near infrared band at 1,040 nm and had a spatial resolution of 1.31 m. Detailed description of specific information concerning bandwidth of the spectral bands and their radiometric characteristics is available [1]. The images were radiometrically calibrated using reference points in overlapping images, processed for geospatial precision using ground control points, and resampled to 3 m spatial resolution. Substantial cloud cover still remained in the data, heavily concentrated in interior highland locations of the island. A maximum NDVI method [2] was used to minimize cloud cover, and a random forest classifier in GEE was used together with the field survey plots obtained for Pohnpei (discussed separately below) as ground training plots. Digital elevation products used in the image classification were derived from Shuttle Radar Topography Mission (SRTM) which was flown in 2000 and resampled at 30 m resolution. Multiple runs of the classifier were conducted to select the best result based on visual inspection of the input imagery. The results were further improved through manual adjustment.

Aerial photographs of Pohnpei were taken in April of 1983. A set of these aerial photographs was purchased by the US Forest Service’s Institute of Pacific Islands Forestry (IPIF) and used by their scientific staff. In 2018, IPIF donated its collection of aerial photographs to the University of Hawaii at Manoa MAGIS program for preservation and use by the public (available at https://guides.library.manoa.hawaii.edu/aerials/digital ) and was digitized from 9-inch square prints (scale 1:12,000). Acquired for this project were 247 color digital aerial photographs along thirty-one flight lines covering the entire mangrove forested areas on all sides of the Pohnpei coast. Processing steps included stripping edges of the digital aerial photographs (which contained various markings) and georectification to WGS 1984, UTM Zone 57N (matching that of the WorldView-3 image projection). To control for quality of the manual process, the root mean squared error (RMSE) of spatial precision was constrained to within 10 m^2^ (average 2 m^2^) by adding between 3 and 21 control points (average 11) for each photo. These photos were mosaicked and used to create an image of the mangrove-occupied area of Pohnpei in 1983.

The island mosaic created from the 1983 aerial photographs was compared to the WorldView-3 mosaic to map mangrove loss and gain using manual interpretation. To do so, the 1983 mosaic was first uploaded to GEE and aligned with the WorldView-3 mosaic and mangrove distribution map. The 2018 mangrove map was then overlaid on the 1983 mosaic and the mangrove edge was visibly traced, with all mangrove pixels in 1983 outside the 2018 mangrove extent manually labeled using GEE’s geometry drawing functionality. These polygons composed the loss class in a mangrove change map. A similar process was used to find mangrove gain, with the 1983 mosaic being displayed at 50% opacity on top of the 2018 mangrove map. The perimeter of the island was manually inspected a total of four times, two to identify mangrove loss and two to identify gain. Finally, the polygons were rasterized to match the dimensions of the input imagery. The result of the change analysis led to a 1983-2018 mangrove loss and gain map for the island of Pohnpei, as shown in Fig 1 in the main text. To account for possible georeferencing errors between the two maps, which could indicate erroneous mangrove gains, shoreline changes less than 10 meters were not considered.

- 1. **Field survey**

Plots were 10 m in radius with 3 m radius subplots within. Species identification, diameter at breast height (DBH) measurements and mortality status (see main paper for more details) were recorded for tall and medium trees (≥ 5 cm DBH) within the larger 10 m radius plots and for saplings (< 5 cm DBH) within the 3 m radius subplots. The DBH measurements were taken at 1.3 m or right above the tallest stilt root at a location not affected by the root junction in the case of *Rhizophora* species. Downed dead wood information was recorded in 236 plots. Downed dead wood ≥ 5 cm in diameter was counted in each plot along four 10 m sampling lines extending from plot center outwards in the four cardinal directions. Due to time constraints, diameter of downed dead wood was not measured but instead was estimated as the average diameter of trees in that plot with diameter ≥ 5 cm [3]; this protocol would result in slight over-estimates as this technique does not account for decomposition of woody debris *in-situ* from the time of debris fall to our survey date.

- 1. **Mapping dominant mangrove species**

Dominant species, defined as species comprising the largest basal area per field plot, were analyzed separately with two geospatial models: k-nearest neighbor (KNN) and random forest (RF), and a common set of predictor variables. Predictor variables with 5 m resolutions were created in ArcMap 10.7.1, including principal components of WorldView-3, WorldView-2 and QuickBird satellite imagery composites; distance from water; elevation; and island side (leeward or windward). The composites were comprised of WorldView-3 eight band images from July, September and October 2018; WorldView-2 three band image from December 2013; and Quickbird four band images from January 2007 and June 2005. To decrease spectral similarities between mangrove species, the images were analyzed using unsupervised principal component analysis (PCA) [4,5]. The first four principal components of the image analysis were used as predictors as they captured 99.55% of the variance. In our model, distance to water and elevation are proxies for unavailable data such as inundation frequency; the salinity gradient and soil physiochemical characteristics such as nitrogen, phosphorous, and sulfide [6,7]. The digital elevation model (DEM) was created by extrapolating elevation data collected on the island (Fig 1) [D. B. Gesch, U.S. Geological Survey, personal communication, September 26, 2019] [8]. It should be noted that the elevation points used to interpolate a DEM surface were extremely sparse, leading to uncertainties in elevation (see Fig 1). Elevation points were collected in clusters, with the minimum and maximum distance between adjacent clusters being 110 m and 16,010 m, respectively. Because of this, subtle changes in elevation could not be modeled. None of the predictors were multicollinear (p<0.05) when tested with the rfUtilities package [9,10] in RStudio [11].

The KNN model was created using the class package in RStudio [12,11]. RF model creation and evaluation were performed using the rfUtilities and randomForest packages in RStudio [9,10,11,13]. Mangrove species models and resulting maps can be found in Peneva-Reed and others [14].

1. **Results supplement**

**3.2. Dominant mangrove species**

The results from the RF model are shown in Fig A in S2 Appendix. *B. gymnorhiza* was predicted to be dominant in 35% of the forest, with all other species dominant in 7% or less of the forest (Table 2). In some areas of the mangrove forest no species were predicted to be dominant. In these areas, species may be more equal in terms of basal area. In other areas, more than one species was predicted to be dominant. This is not surprising since these species were modeled separately and because they have much overlap in the range of predictors in which they can survive and thrive. All RF models were significant at a 95% confidence interval (p<0.05) (Supporting Information: Table B). Once the models were created for each species, the model algorithms were used to “back predict” the data (or to find the predicted species dominance at field plots). This initial model evaluation was good for *B. gymnorhiza*, *R. apiculata*, *R. mucronata*, *S. alba* and *X. granatum* (Supporting Information: Table B). However, *L. littorea*, *R.* x *lamarckii*, and *R. stylosa* had poor area under the **receiver operating** **characteristics curve (AUC) values meaning the model had difficulty distinguishing between dominant and nondominant locations for these species.** *L. littorea* and *R.* x *lamarckii* **also had low** Cohen's Kappa values from back predicting indicating only moderate agreement with field data. Upon further inspection of the models, a 10-fold, 1,000-time cross-validation yielded low cross-validation kappas ranging between 0 and 0.34 for all models, which equates to no agreement to fair agreement. Cross-validation kappas may be low due to imbalances between the numbers of dominant and nondominant plots despite the precautions that were used to minimize this effect. The relatively high RF model performance after back prediction compared with the fair to no agreement according to cross-validation kappas tell us it is likely that our RF models were overfitted.

**Disclaimer**

Any use of trade, firm, or product names is for descriptive purposes only and does not imply endorsement by the U.S. Government.

**S1 Appendix references**

1. Vajsová B, Walczynska A, Aastrand P, Barisch S, Hain S. New sensors benchmark report on WorldView-3. EUR 27673 EN. Luxembourg: Publications Office of the European Union **2015**, doi:10.2788/237561
2. Holben BN. Characteristics of maximum-value composite images from temporal AVHRR data. *Int. J. Remote Sens*. **1986**, 7: 1417-1434.
3. Peneva-Reed EI, Krauss KW, Bullock EL, Zhu Z, Woltz VL, Drexler JZ, ... Stehman SV. Carbon stock losses and recovery observed for a mangrove ecosystem following a major hurricane in Southwest Florida. *Estuar. Coast. Shelf Sci.* **2020**, in press
4. Abdollahnejad, A.; Panagiotidis, D.; Joybari, S.S.; Surový, P. Prediction of dominant forest tree species using quickbird and environmental data. *Forests* **2017**, 8: 42.
5. Liu, L.; Coops, N.C.; Aven, N.W.; Pang, Y. Mapping urban tree species using integrated airborne hyperspectral and LiDAR remote sensing data. *Remote Sens. Envir*. **2017**, 200: 170-182.
6. Crase, B.; Liedloff, A.C.; Wintle, B.A. A new method for dealing with residual spatial autocorrelation in species distribution models. *Ecography* **2012**, 35: 879-888.
7. McKee, K.L.; Feller, I.C.; Popp, M.; Wanek, W. Mangrove isotopic (δ^15^N and δ^13^C) fractionation across a nitrogen vs. phosphorus limitation gradient. *Ecology* **2002**, 83: 1065-1075.
8. Thorne, K.; Buffington, K.M.; MacKenzie, R.A.; Krauss, K.; Ellison, J.C.; Peneva-Reed, E.; et al. Modeling mangrove ecosystem sea-level rise vulnerability for Pohnpei, Micronesia [abs.]. In Fall Meeting, San Francisco, California. Proceedings: Washington, D.C., American Geophysical Union **2019**, pap. GC51K-0995
9. Evans, J.S.; Murphy, M.A.; Holden Z.A.; Cushman, S.A. Modeling species distribution and change using random forest. In *Predictive species and habitat modeling in landscape ecology: Concepts and applications*. Drew C, Wiersma Y, Huettmann F, eds. New York: Springer Science and Business Media. **2011**, P139-159.
10. Evans, J.S.; Murphy, M.A. Random forests model selection and performance evaluation. https://cran.r-project.org/. [R package version 2.1–5]. **2019.**
11. RStudio Team. RStudio: Integrated Development for R. RStudio, Inc., Boston, MA URL <http://www.rstudio.com/>. **2018.**
12. Ripley, B.; Venables, W. Functions for classification https://cran.r-project.org/. [R package version 7.3-15]. **2019**.
13. Breiman, L.; Cutler, A.; Liaw, A.; Wiener, M. Breiman and Cutler's random forests for classification and regression. https://cran.r-project.org/. [R package version 4.6–14]. **2018.**
14. Peneva-Reed EI, Woltz VL, and Zhu Z. Aboveground mangrove biomass data collected from and species dominance maps of in 2016-17 from Pohnpei, Federated States of Micronesia. U.S. Geological Survey data release. 2019. Available from: https://doi.org/10.5066/P9JAE5JC
