# Supplementary material for: A comprehensive assessment of mangrove species and carbon stock on Pohnpei, Micronesia
Source: PLoS One. 2022 Jul 21;17(7):e0271589. doi: 10.1371/journal.pone.0271589 (PMC9302747; doi:10.1371/journal.pone.0271589)
Supplement: S2 Appendix — (DOCX) [file pone.0271589.s002.docx]

**S2 Appendix**

**Tables** **supplement**

**Table A. Allometric equations for Mangroves.**

| **Species** | **Equation** | **Reference** |
| --- | --- | --- |
| *B. gymnorhiza* | *B_T_* = *B_S1_* + *B_L_* |  |
|  | *B_S1_* = 0.0483 * (*D*^2.589) * *Sg* | [1,2] |
|  | *B_S3_* = 0.0473 * (*D*^2.539) * *Sg* | [1,2] |
|  | *B_L_* = 10^(-1.1679 + 1.4914 * Log(*D*)) | [3] |
| *L. littorea* | *B_T_* = *B_S1_* / 0.975 | [4] |
|  | *B_S1_* = 0.0796 * (*D*^2.456) * *Sg* | [1,2] |
|  | *B_S3_* = 0.0883 * (*D*^2.351) * *Sg* | [1,2] |
|  | *B_L_* = *B_T_* * 0.025 | [4] |
| *R. apiculata*^a^ | *B_T_* = *B_S1_* + *BL* + *B_R_* |  |
|  | *B_S1_* = 0.0579 * (*D*^2.692) * *Sg* | [1,2] |
|  | *B_S3_* = 0.0792 * (*D*^2.546) * *Sg* | [1,2] |
|  | *B_L_* =10^(1.378 * Log(*D* * π) - 1.632) | [5] |
|  | *B_R_* = 10^(2.546 * Log(*D* * π) - 2.94) | [5] |
| *R, stylosa*^b^ | *B_T_* = 10^(-0.9789 + 2.6848 * Log(*D*)) | [3] |
|  | *B_S1_* = *B_T_* - *B_L_* |  |
|  | *B_S3_* = 10^(-1.0528 + 2.5621 * Log(*D*)) | [3] |
|  | B_L_ = 10^(-1.8571 + 2.1072 * Log(*D*)) | [3] |
|  | B_R_ = 10^(-2.1663 + 3.1353 * Log(*D*)) | [3] |
| *R.* species unknown | *B_T_* = 10^(-0.9789 + 2.6848 * Log(*D*)) | [3] |
|  | *B_S1_* = *B_T_* – *B_L_* |  |
|  | *B_S3_* = 10^(-1.0528 + 2.5621 * Log(*D*)) | [3] |
|  | *B_L_* = 10^(-1.8571 + 2.1072 * Log(*D*)) | [3] |
|  | *B_R_* = 10^(-2.1663 + 3.1353 * Log(*D*)) | [3] |
| *S. alba* | *B_T_* = *B_S1_* + *B_L_* |  |
|  | *B_S1_* = 0.128 * (*D*^2.359) * *Sg* | [1,2] |
|  | *B_S3_* = 0.0751 * (*D*^2.458) * *Sg* | [1,2] |
|  | *B_L_* = 10^(0.1616 - 0.3179 * log(*D*)) | [6] |
| *X. granatum* | *B_T_* = *B_S1_* + *B_L_* |  |
|  | *B_S1_* = 0.187 * (*D*^2.249) * *Sg* | [1,2] |
|  | *B_S3_* = 0.149 * (*D*^2.249) * *Sg* | [1,2] |
|  | *B_L_* = 10^(-2.2380 + 2.3966 * Log(*D*)) | [1,2] |

Allometric equations used to compute biomass components of mangrove species given diameter at breast height. Where B is biomass in kg of whole trees; dead status one and three trees; leaves; and prop roots when superscript T; S1, S3; L; and R are used, respectively. Biomass was calculated in dead status one trees by subtracting the biomass of the leaves from the whole tree, status two by subtracting 15% of the whole tree biomass, and status three as the biomass of only the main stem [4]. D is diameter in centimeters at breast height, or above the highest prop root in the case of Rhizophora species, and Sg is specific gravity in g/cm^3^.

a Allometric equations derived for *R. apiculata* were also used for *R. x lamarckii* because *R. x lamarckii* is a hybrid between *R. apiculata* and *R. stylosa*.

b Allometric equations derived for *R. stylosa* were also used for *R. mucronata* because they are most similar in form among the *Rhizophora* species sampled.

**Table B. Model evaluation.**

| **Species** | **Significance** | **PCC** | **Cohen's Kappa** | **AUC** | **TSS** |
| --- | --- | --- | --- | --- | --- |
| *B. gymnorhiza* | 0 | 100% | 1 | 1 | 1 |
| *L. littorea* | 0.001 | 91% | 0.35 | 0.62 | 0.9 |
| *R. apiculata* | 0.001 | 99% | 0.96 | 0.97 | 0.98 |
| *R. x lamarckii* | 0.001 | 92% | 0.41 | 0.64 | 0.92 |
| *R. mucronata* | 0.001 | 93% | 0.64 | 0.76 | 0.93 |
| *R. stylosa* | 0.001 | 76% | 0.82 | 0.53 | 0.76 |
| *S. alba* | 0.001 | 95% | 0.86 | 0.9 | 0.94 |
| *X. granatum* | 0.001 | 92% | 0.7 | 0.79 | 0.91 |

Random forest model significance and percent correctly classified (PCC), Cohen’s Kappa, Area under the receiver operator characteristics curve (AUC) and the true skill statistic (TSS) based on the back predicted data.

**Table C. Community composition by location on Pohnpei.**

|  |  | **ISLAND SIDES** | | **ZONES** | | |
| --- | --- | --- | --- | --- | --- | --- |
| **Species** | **Variable** | **Leeward** | **Windward** | **Seaward** | **Interior** | **Landward** |
| *B. gymnorhiza* | Basal area | 10.3 ± 0.9 | 9.4 ± 1.2 | 5.3 ± 1.5 ^c,d^ | 10.5 ± 0.8 ^b^ | 12.0 ± 2.2 ^b^ |
|  | Density | 419 ± 38 | 205±25 | 267 ± 76 | 388 ± 33 | 202 ± 35 |
|  | IVI % | 34 | 25 | 28 | 32 | 28 |
| *L. littorea* | Basal area | 1.3 ± 0.5 | 3.6 ± 2.0 | 0.0 ± 0.0 ^c^ | 2.4 ± 1.0 ^b^ | 2.7 ± 1.6 |
|  | Density | 15 ± 5 | 21±10 | 0 ± 0 | 18 ± 6 | 25 ± 16 |
|  | IVI % | 3 | 4 | 0 | 4 | 5 |
| *R. apiculata* | Basal area | 5.4 ± 0.6 ^a^ | 7.6 ± 0.8 ^a^ | 7.4 ± 1.5 | 5.9 ± 0.5 | 6.2 ± 1.2 |
|  | Density | 250 ± 25 | 281±28 | 338 ± 55 | 245 ± 22 | 262 ± 56 |
|  | IVI % | 22 | 28 | 35 | 22 | 27 |
| *R. stylosa/ mucronata* | Basal area | 3.5 ± 0.6 | 2.6 ± 1.0 | 3.6 ± 1.6 | 3.3 ± 0.5 | 1.9 ± 0.8 |
|  | Density | 117 ± 23 | 56 ± 21 | 120 ± 50 | 105 ± 20 | 29 ± 10 |
|  | IVI % | 12 | 8 | 16 | 11 | 16 |
| *R. x lamarckii* | Basal area | 0.6 ± 0.2 | 0.7 ± 0.5 | 1.3 ± 0.8 | 0.6 ± 0.2 ^d^ | 0.0 ± 0.0 ^c^ |
|  | Density | 68 ± 19 | 52 ± 32 | 61 ± 37 | 75 ± 22 | 1 ± 1 |
|  | IVI % | 4 | 4 | 7 | 4 | 0 |
| *S. alba* | Basal area | 4.9 ± 0.8 ^a^ | 21.7 ± 3.5 ^a^ | 4.5 ± 2.1 ^c^ | 11.4 ± 1.7 ^b^ | 13.1 ± 3.7 |
|  | Density | 27±4 | 102 ± 16 | 64 ± 29 | 55 ± 7 | 28 ± 7 |
|  | IVI % | 9 | 27 | 12 | 16 | 15 |
| *X. granatum* | Basal area | 8.8 ± 1.6 ^a^ | 2.0 ± 0.9 ^a^ | 0.2 ± 0.1 ^c,d^ | 6.2 ± 1.2 ^b^ | 14.3 ± 5.1 ^b^ |
|  | Density | 73±13 | 13 ± 4 | 5 ± 3 | 58 ± 12 | 69 ± 21 |
|  | IVI % | 15 | 4 | 2 | 11 | 18 |
| Total | Basal area | 34.8* ± 2.0 | 47.7* ± 3.9 | 22.2* ± 2.3 | 40.4* ± 2.3 | 50.1* ± 5.4 |
|  | Density | 972* ± 43 | 732* ± 40 | 885 ± 69 | 947* ± 40 | 621* ± 57 |
|  | IVI % | 100* | 100 | 100 | 100 | 100* |

Mean basal area (m^2^ ha^-1^) and tree density (trees ha^-1^) ± standard error and importance value index as a % for mangroves occupying in different areas on Pohnpei. *Values do not add up because totals include unidentified Rhizophora species and/or because of rounding. T-tests were used to compare each species’ basal area on island sides (with ^a^ indicating a significant difference between sides) and zones (with ^b^, ^c^ and ^d^ indicating a significant differences between seaward, interior and landward zones, respectively) (p<0.05).

**Figures supplement**


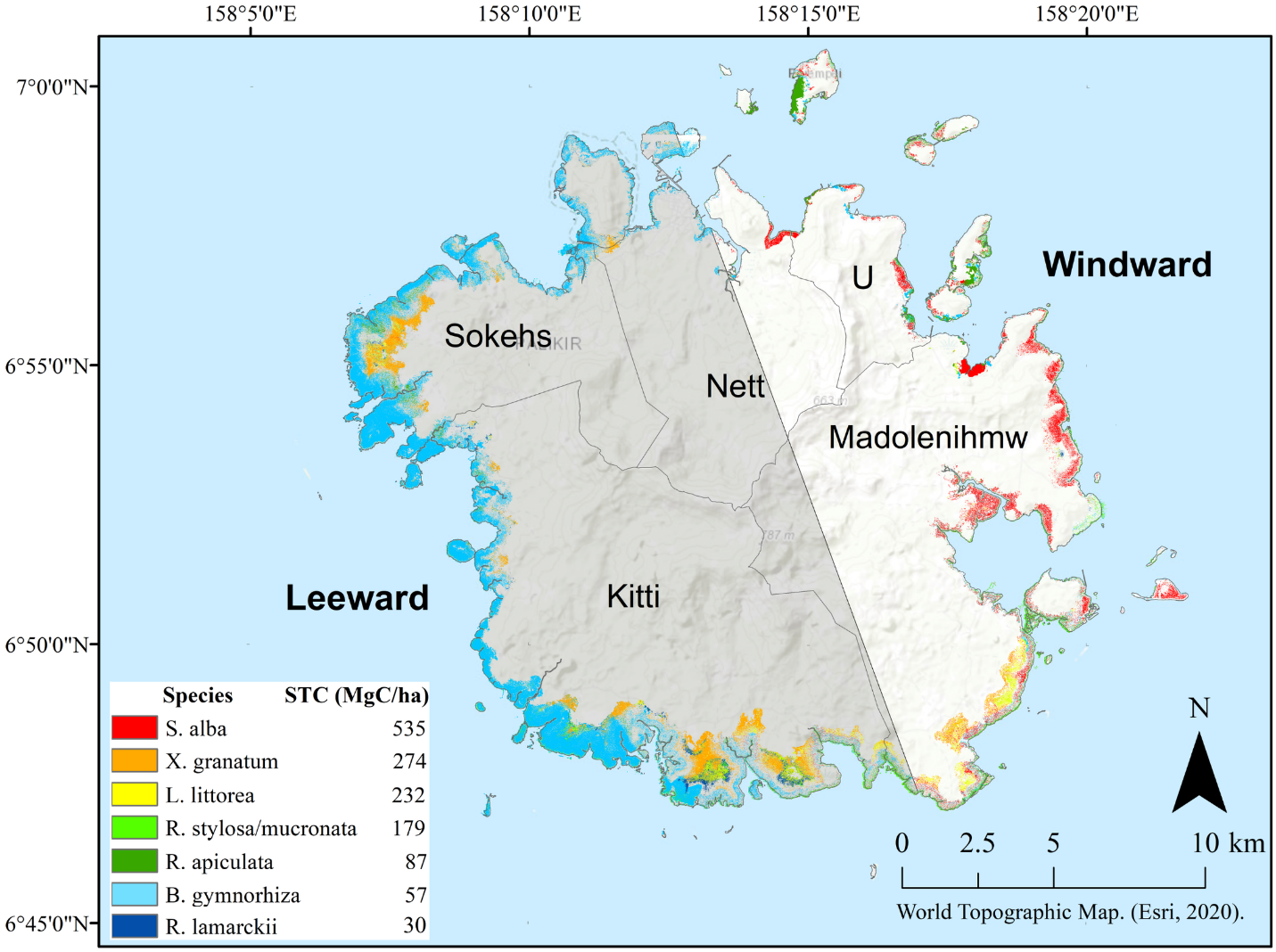


**Fig A. Predicted mangrove dominance.** Dominant mangrove species and associated standing tree carbon (STC) stock (MgC ha^-1^) in 5 by 5 meter cells mapped using the random forest method, overlaid on Esri’s topographic basemap of the island of Pohnpei [7]. Municipality names and windward and leeward island sides (translucent white and grey, respectively) are also shown on the map.

**Disclaimer**

Any use of trade, firm, or product names is for descriptive purposes only and does not imply endorsement by the U.S. Government.

**S2 Appendix references**

1. Cole, T.G.; Ewel, K.C.; Devoe, N.N. Structure of mangrove trees and forests in Micronesia. *For. Ecol. Manag.***1999**, 117: 95-109.
2. Kauffman, J.B.; Cole, T.G. Micronesian mangrove forest structure and tree responses to a severe typhoon. *Wetlands* **2010**, 30: 1077-1084.
3. Clough BF, Scott K. Allometric relationships for estimating above-ground biomass in six mangrove species. For. Ecol. Manag. **1989**, 27: 117-127.
4. Kauffman, J.B.; Donato, D.C. Protocols for the measurement, monitoring and reporting of structure, biomass, and carbon stocks in mangrove forests. Working Paper 86. Bogor, Indonesia: CIFOR. **2012**, p40.
5. Ong, J. E., Gong, W. K., & Wong, C. H. Allometry and partitioning of the mangrove, Rhizophora apiculata. *For. Ecol. Manag.* **2004**, 188(1-3), 395-408.
6. Kairo JG, Bosire J, Langat J, Kirui B, Koedam N. Allometry and biomass distribution in replanted mangrove plantations at Gazi Bay, Kenya. *Aquat. Conserv.* **2009***,* 19: S63-S69.
7. Esri. World Topographic Map. Sources: Esri, HERE, Garmin, Intermap, increment P Corp., GEBCO, USGS, FAO, NPS, NRCAN, GeoBase, IGN, Kadaster NL, Ordnance Survey, Esri Japan, METI, Esri China (Hong Kong), (c) OpenStreetMap contributors, and the GIS User Community. **2020.**
